# Supplementary material for: Association between oral microbiome and seven types of cancers in East Asian population: a two-sample Mendelian randomization analysis
Source: Front Mol Biosci. 2023 Nov 21;10:1327893. doi: 10.3389/fmolb.2023.1327893 (PMC10702768; doi:10.3389/fmolb.2023.1327893)
Supplement: Supplementary file 1 [file DataSheet1.ZIP › Supplementary Materials/MR plots of saliva/saliva═╝/Prostate cancer/pheno.3088_to_prostate cancer_forest.pdf]

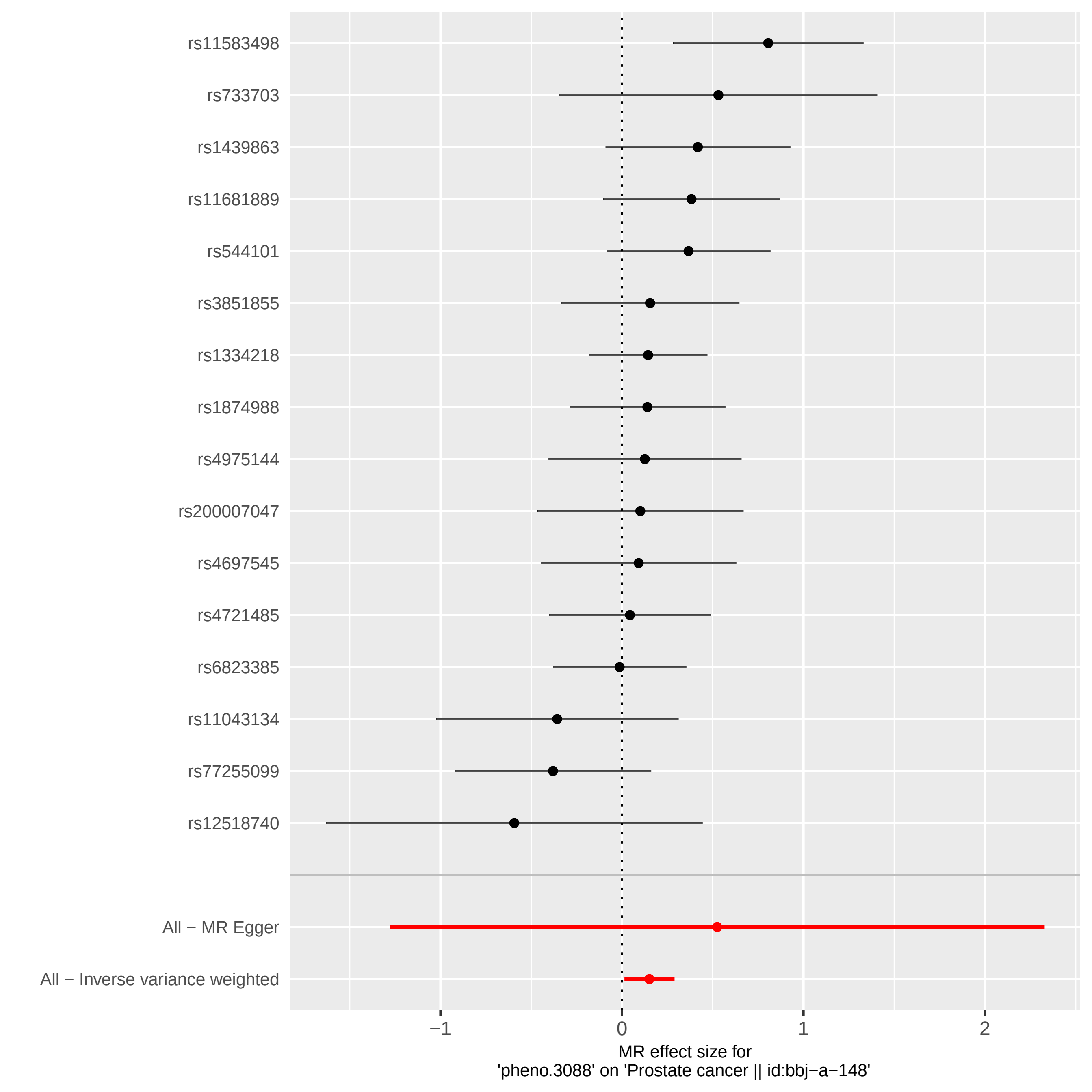
rs11583498
rs733703
rs1439863
rs11681889
rs544101
rs3851855
rs1334218
rs1874988
rs4975144
rs200007047
rs4697545
rs4721485
rs6823385
rs11043134
rs77255099
rs12518740
All − MR Egger
All − Inverse variance weighted
−1
0
1
2
MR effect size for
'pheno.3088' on 'Prostate cancer || id:bbj−a−148'
